# Impact of Québec’s healthcare reforms on the organization of primary healthcare (PHC): a 2003-2010 follow-up

**DOI:** 10.1186/1472-6963-14-229

**Published:** 2014-05-21

**Authors:** Raynald Pineault, Roxane Borgès Da Silva, Alexandre Prud’homme, Michel Fournier, Audrey Couture, Sylvie Provost, Jean-Frédéric Levesque

**Affiliations:** 1 Direction de santé publique de l’Agence de la santé et des services sociaux de Montréal, Montréal, QC, Canada; 2 Institut national de santé publique du Québec, Québec, QC, Canada; 3 Centre de recherche du Centre hospitalier de l’Université de Montréal, Montréal, QC, Canada; 4 Institut de recherche en santé publique de l’Université de Montréal, Montréal, QC, Canada; 5 Faculté des sciences infirmières de l’Université de Montréal, Montréal, QC, Canada; 6 Centre for Primary Health Care and Equity, University of New South Wales, New South Wales, Australia

**Keywords:** Primary healthcare organization, Healthcare reform, Organizational change, Family practice

## Abstract

**Background:**

Healthcare reforms initiated in the early 2000s in Québec involved the implementation of new modes of primary healthcare (PHC) delivery and the creation of Health and Social Services Centers (HSSCs) to support it. The objective of this article is to assess and explain the degree of PHC organizational change achieved following these reforms.

**Methods:**

We conducted two surveys of PHC organizations, in 2005 and 2010, in two regions of the province of Québec, Canada. From the responses to these surveys, we derived a measure of organizational change based on an index of conformity to an ideal type (ICIT). One set of explanatory variables was contextual, related to coercive, normative and mimetic influences; the other consisted of organizational variables that measured receptivity towards new PHC models. Multilevel analyses were performed to examine the relationships between ICIT change in the post-reform period and the explanatory variables.

**Results:**

Positive results were attained, as expressed by increase in the ICIT score in the post-reform period, mainly due to implementation of new types of PHC organizations (Family Medicine Groups and Network Clinics). Organizational receptivity was the main explanatory variable mediating the effect of coercive and mimetic influences. Normative influence was not a significant factor in explaining changes.

**Conclusion:**

Changes were modest at the system level but important with regard to new forms of PHC organizations. The top-down decreed reform was a determining factor in initiating change whereas local coercive and normative influences did not play a major role. The exemplar role played by certain PHC organizations through mimetic influence was more important. Receptivity of individual organizations was both a necessary condition and a mediating factor in influencing change. This supports the view that a combination of top-down and bottom-up strategy is best suited for achieving substantial changes in PHC local organization.

## Background

### Informational background

In the early 2000s, Québec initiated important reforms of its healthcare system, involving more particularly its primary healthcare (PHC) sector. New modes of PHC delivery were introduced, as Family Medicine Groups (FMGs) were created [1,2]. An FMG consists of 6 to 10 physicians who work with nurses, to provide services to enrolled patients on a non-geographical basis (between 10,000 to 15,000 people per FMG). It offers increased accessibility through extended opening hours and participation in a regional on-call system. The target established at the onset of the reform was to have 300 FMGs in the province of Québec. As of November 2013, there were 255 accredited FMGs in the province, 41 in Montréal and 38 in Montérégie, the two regions participating in this study. An FMG can be affiliated with more than one setting, since it is based on physicians’ participation and engagement, regardless of their practice setting. Hence, for one FMG there is one main setting, but there may be several affiliated ones. As of November 2013, in the two regions under study, there were 79 primary and 84 affiliated settings, for a total of 79 FMGs, but 163 participating settings. A complementary model of PHC organizations, implemented in the two regions but mainly in Montréal under the initiative of the Regional Health Agency, was the Network Clinic (NC). These clinics are more specifically aimed to foster accessibility through walk-in visits and provide access to technical support, such as X-rays and lab tests, and to specialists. The distinction between FMG and NC is often blurred, as many clinics have gained both status, and thus benefit from two sources of funding, provincial and regional. As of November 2013, 32 PHC organizations had the double FMG-NC status in Montréal and Montérégie, whereas 8 had only NC status and 131 only FMG status.

A local coordination structure was also created, with 95 Health and Social Services Centers (HSSC) being entrusted by the Ministry to create Local networks of services. These HSSCs were to foster better coordination among PHC services providers and between PHC and specialized services. They were created by law, merging on a geographical basis long-term care hospitals, Local Community Services Centers (LCSCs) and, in most cases, acute care hospitals. LCSCs are public clinics created in the early 1970s, under the governance of the provincial government. They provide health and social services. Aside from merging these organizations, the main responsibility of HSSC was to increase collaboration among local actors within local networks and consequently, to support the development of new emerging forms of organizing PHC at the local level. The organization of PHC thus constitutes a core element in the establishment of local networks. A few years after implementation of these reforms, it is legitimate to assess their impact particularly on the organization of PHC services and to identify the local contribution of contextual factors that can facilitate or hinder these changes. These are the issues addressed in this article.

### Theoretical background

The literature on organizational change is abundant and covers a wide array of related topics from different theoretical perspectives [3-7]. One review focusing on healthcare attempts to draw lessons on what has been learned about organizational change in the last two decades regarding both vertical and horizontal integration [8]. This review, along with other articles, highlights four recurring themes in the literature on organizational change: content of change, namely organizational characteristics that affect the scope and magnitude of change; contextual factors, such as internal receptivity and external pressures; processes and dynamics involved in implementing changes, such as timing, pace and sequencing of events; and criterion issues, related mainly to outcomes and consequences of change [6,8,9].

Despite the abundance of publications on the subject, specific research deficiencies are underscored by several authors. First, there are still few longitudinal research studies that connect explanatory contextual factors and consequences of change [6,9,10]. Authors also point to the paucity of research that looks at multiple contexts and levels of analysis and that attempts to systematically assess content, context, process and outcomes [6]. Finally, not enough attention has been given to receptivity and readiness of organizations as factors influencing implementation of change [9,11,12].

Organizational change is not an instantaneous event but rather a process that unfolds over time [13]. Following the conceptual work of Pettigrew et al. [9], Newton et al. [12] defined receptive and non-receptive organizations in terms of their variability in the rate, pace and sequencing of changes adopted. As the process of change unfolds over time, features of receptivity can be temporally ordered. For example, FMGs and NCs were completely free to initiate the process that led to their taking on these new organizational forms. The implementation process followed a sequence of events: intention and application to become FMG or NC, acceptance by the Ministry and/or Regional Agency and accreditation. While accreditation is a critical step, the process of change does not stop there but continues to unfold over time, leading to changes in structures, resources and practices.

With respect to content of change, it is possible to construct a measure of organizational change based on the configurational approach to organization. Meyer et al. [14] advocated the configurational approach as a better way to grasp organizations in their entirety and complexity, by representing them as a set of coherent, closely interrelated attributes, rather than individual and independent attributes. Greenwood and Hinings [15] have convincingly argued that organizations are better understood by analyzing overall patterns than by analyzing narrowly drawn sets of organizational properties. They argue in favor of using archetypes and the configurational approach to analyze organizational change. An archetype is thus a set of organizational properties that consistently embodies a single interpretive scheme [15]. One way to operationalize this approach is to compare an organization to an ideal type theoretically derived from the literature and to measure its conformity or proximity to this ideal type [16,17]. This index of conformity to an ideal type (ICIT) can then be used to assess the content change in organizations over time.

The construction of an ideal type rests upon a theoretical and conceptual foundation. The configurational approach provides part of this foundation. There is no single generally accepted definition of what an organization is. An organization is essentially a social arrangement for collective action, where activities are planned and coordinated to achieve its mission and attain its goals [18,19]. To accomplish its mission, an organization requires resources that can be mobilized to produce services, a structure of governance that guides its members in carrying out their activities, as well as exchanges with its environment. Consequently, attributes of an organization are related to four domains: (1) a mission that states its goals and orientations; (2) a structure that sets a regulatory and governance framework for action; (3) resources that are required to produce services; and (4) professional and administrative practices embedded in mechanisms that support the production and delivery of services. Furthermore, in each of these four domains, organizations must develop and maintain exchanges with their specific environments. For example, in setting its goals, an organization must consider if they adequately meet the higher level of goals of the healthcare system. Likewise, sharing resources and establishing collaborations among organizations enable them to widen the scope and improve the quality of services offered.

An organization can change over time in response to various influences and processes exerted by its environment. According to the institutional theory, organizations conform to contextual pressures to gain legitimacy and to increase their chances of survival [20]. These influences can be of three types: coercive, normative and mimetic [20,21]. Coercive influences refer to laws, regulative rules and incentives. In public systems, the state and its subsidiaries have the definite ability to apply and enforce such mechanisms. Normative influences refer to values and norms shared by professionals and their associations. This type of influence tends to permeate organizational boundaries [22]. Finally, mimetic influences rest upon examples of what could and should be attained. They provide a common framework of meaning and sense making for actions [20]. Examples of models to imitate are often provided by institutional leaders and early adopters of innovation [21].

These three influences, particularly when they converge, are potent levers for change. The ultimate result is a form of isomorphism characterized by a high degree of homogeneity of organizations within an institutional field or sector in which diverse actors share common norms and values in response to the three types of influences to which they are exposed [20,21,23]. However, the pressure exerted by the three types of influence does not necessarily affect all organizations equally, thus limiting the attainment of isomorphism. Individual organizations respond and react differently to these pressures. Intrinsic characteristics of organizations, such as size, type of leadership and organizational culture make organizations more or less responsive and in some extreme cases, even resistant to extraneous influences leading eventually to a form of inertia [15,21]. This property called receptivity is very often present among early adopters of innovations [9,21]. The more receptive an organization, the more responsive it will be to pressures for change and innovation. As a corollary, the more receptive organizations there are in an institutional sector, the higher the degree of isomorphism can be attained in that sector.

### Conceptual framework

**Figure 1:**
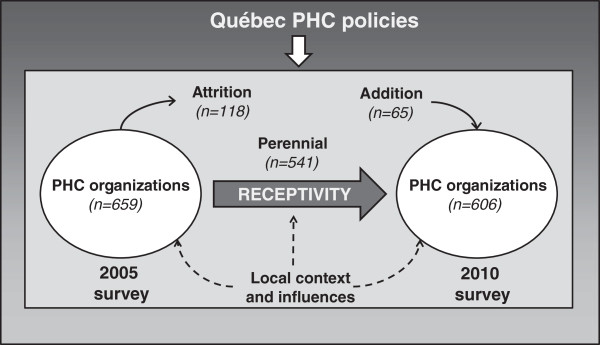
Conceptual framework.

Based on the elements just presented, the conceptual framework of our study posits that overall Québec’s healthcare reform induced PHC organizational changes and that this influence was reinforced or deterred by contextual characteristics of local networks, as well as by influences exerted within the local networks by HSSCs, professional organizations and other PHC organizations [24]. As pointed out earlier, organizations respond differently to these influences, due to intrinsic and idiosyncratic characteristics that create different degrees of receptivity to change, which in turn mediates the effect these influences have on change. Organizational change is expressed by the difference in the ICIT scores between 2003 and 2010 for perennial organizations that were present both in the 2005 and 2010 surveys. Scores of FMGs accredited prior to 2005 were adjusted to take into account the changes likely to have occurred between accreditation time and the 2005 survey. Further details about adjustment are provided in the Methods section. The analysis also takes into account organizations that closed (attrition) and that opened (addition) between the 2005 and 2010 surveys. These variables and their relationships are shown in Figure 1.

Our study is intended as a response to less explored areas of research on organizational change in healthcare identified by authors. First, we chose a longitudinal strategy by looking at two points in time. We define organizational change as an empirical observation of differences in forms, states and properties that characterize an organizational entity over time [13]. Second, we documented the extent of change by looking at different components of organizations involved. Finally, we adopted a multilevel strategy to look at the intra-organizational and contextual characteristics and processes.

### Objectives

The objectives of this article are: (1) to assess the extent of organizational change associated with the Québec reform processes between 2003 and 2010, and the extent to which the new organizational forms (FMGs and NCs) have contributed to observed change, measured by the index of conformity to an ideal type (ICIT), as well as organizations that closed and that opened during that period; (2) to determine the importance of contextual factors, local influences and factors associated with receptivity when explaining observed change in the index of conformity to an ideal type (ICIT).

## Methods

### Research design

We conducted two surveys of PHC organizations (2005 and 2010) in the two most populous regions of the province of Québec, Montréal and Montérégie [24]. The 2010 mail questionnaire was mainly based on the 2005 one, with particular attention paid to comparability of the questions, so as to make valid comparisons between the two time periods. In the 2010 questionnaire, a section was added seeking retrospectively information on various aspects of the reform and its impact on the organizations surveyed. The other sections of the questionnaire covered the four organizational domains presented earlier: vision, structure, resources and practices (Additional file 1).

The questionnaire was first developed in the 2005 study. It was tested for face validity to assess the relevance of the questions it contains. It was also tested for content validity to determine the degree of exhaustivity of the questions covering the concepts. Because of the nature of the indicators derived from the questionnaires, a formative approach to index construction was preferred to the classic reflective approach to scale development and validation. In the reflective approach, items composing a scale are effect indicators because they all reflect the latent construct and are necessarily correlated. In the formative approach, items are causal indicators contributing to form the composite index and they may not be correlated [25-27]. Consequently, factor analysis and Cronbach’s alpha coefficients are not appropriate statistical tools for validating a composite index and were not used in our study. An additional reason for not using these tools is that responses to questions are in the form of reporting rather than rating.

A total of 472 PHC organizations participated in the 2005 survey for a 72% response rate (67% in Montréal and 81% in Montérégie). In 2010, 376 PHC organizations participated in the survey for a 62% response rate (60% in Montréal and 66% in Montérégie). The various types of PHC organizations were well represented (FMGs-NCs, FMGs, NCs, LCSCs and other clinics). As pointed out earlier, FMG-NC, FMG and NC practices included main and affiliated settings. A key informant identified in each clinic, usually the doctor responsible for the professional and/or administrative matters of the practice, responded to the mail questionnaire. To draw inferences on all PHC organizations and to assess the impact of the healthcare reform on the whole system, we needed to include all the organizations. We thus applied an imputation technique to non-responding organizations based on the probability of their responses, given the region, type and size of the group of organizations to which they belonged [28]. Details of these procedures and calculation are available from the authors, upon request.

This study was performed according to the principles of the Helsinki Declaration. The Research Ethics Committee of the Agence de la santé et des services sociaux de Montréal approved the study. Participants had to sign an informed consent and were told that they could withdraw during the study.

### The ICIT score

As pointed out earlier, organizational change was assessed by comparing the index of conformity to an ideal type (ICIT) in 2003 and 2010. The index includes 26 attributes, corresponding to selected responses to the organizational questionnaire and distributed in the four domains described previously (Additional file 2). The choice of the 26 attributes was guided by recent literature and proposals promoting the implementation of the patient-centered medical home concept into PHC [29-32]. An organization that possessed an attribute received a score of 1 or 2, depending whether the variable was on a two- or three-point scale and a score of 0 if it did not possess the attribute. The total maximum score was 52. The score was then expressed on a 100-point scale to facilitate interpretation.

### Measure of organizational change

Since ICIT scores observed in 2005 underestimated the changes that had occurred for FMGs accredited prior to 2005, we adjusted the scores of these FMGs. Using a regression analysis model, we calculated changes in ICIT scores as a function of the time elapsed since accreditation for FMGs and NCs accredited after 2005. The rate of change increased steadily during the first 30 months and then leveled off (data not shown). We then applied these figures to the 48 FMGs accredited prior to 2005, which represent 8.9% of perennial PHC organizations, based on the number of months elapsed between their accreditation and the 2005 survey. The assumption is that the rate of change for FMGs prior to 2005 was similar to those accredited after 2005. This is a sound assumption, given the fact that eligibility criteria for FMGs have remained relatively stable since the onset of the reform. For the other PHC organizations, we made the assumption that they had not changed much in the period prior to 2005, more specifically in the two years prior to the 2005 survey. This is also a sound assumption, since we observed very minimal changes in PHC organizations other than FMGs and NCs during the post-reform period, extending from March 2003, which is the date of the first FMG’s accreditation, to April 2010, which is the time of the second survey.

Overall organizational change at the system level was measured by the difference between average ICIT score of all organizations existing in 2010 and those in 2005 to which their estimated pre-reform score was attributed. The difference is the results of three effects. The first is the effect of change in perennial organizations. The second is the addition effect attributable to new organizations created during the post-reform period and was obtained by calculating the difference in ICIT scores, with and without these organizations. The third effect is due to attrition of the clinics that closed, merged or ceased to operate in their current form during the post-reform period (attrition effect). This effect was measured by calculating what the ICIT score would have been in 2010 if these clinics had then been in operation and subtracting it from the current ICIT score, assuming that the clinics would not have changed otherwise during that period of time.

Since the size of an organization can influence the total supply of services it provides, and consequently has an effect on the system, we weighted the ICIT score by the clinic size, as measured by the number of full-time equivalent physicians, in the descriptive analyses and the multiple regression analyses. We defined a full-time equivalent physician as one providing 26 hours or more of clinical activities weekly in that clinic, based on expert opinions. The number does not include the time devoted to clinical activities in other settings.

### Explanatory variables

There are two levels of explanatory variables: organizational and contextual (local network). The main organizational variable is receptivity. As pointed out earlier, receptivity of organizations is conceptualized as variability in the rate, sequencing and pace of changes adopted in response to coercive, normative, and mimetic environmental pressures. Accordingly, the process of receptivity unfolds over time from intention to become a new model of PHC service delivery to the critical event of accreditation and the time that follows accreditation. This variable included three categories: whether an organization had acquired FMG, NC, or the two status (FMG-NC); (was not FMG or NC in 2010 but expressed the desire to become one of these or both if it could; and finally, did not want to become either FMG or NC. This variable was meant to be categorical, as FMGs and NCs are considered to be receptive to change, since they were “early adopters” and certainly motivated to espouse this innovative approach. The second category represents those that are not yet FMG or NC, but would like to become one or both of these, if they could. The third category consists of those not interested in changing their current status to become FMG or NC. These three categories were coded as dummy variables in the analysis (Additional file 3).

The second set of organizational indicators at the organizational level was perceived coercive, normative and mimetic influences exerted on PHC organizations. Obviously, this information was available only for organizations in operation in 2010 (perennial). A score for each indicator was constructed by combining responses to the question “How would you assess the effect of the following factors on the evolution of your clinic?”. The effects could be positive, null or negative and were given a score of 2, 1 or 0, accordingly. Details of the items and the construction of the indices are presented in Additional file 3. Coercive, normative and mimetic influences were also assessed at the local network’s level, by calculating the percentage of organizations that assessed as positive the influence of these factors on the evolution of their organization. Correlations between organizational and local network variables were sufficiently low (.37, .32 and .35) to warrant their use while preventing endogeneity. Other organizational and local network indicators were used as control variables (Additional file 4).

### Statistical analysis

The analysis was performed in two stages. First, descriptive statistics on organizational change are presented. Second, explanatory and control variables are entered into two-level models of analysis according to an analytical framework to be presented in the following section, using HLM (version 6.0) software. Considering that organizations are nested within HSSC territories, generalized linear mixed models (GLMM) with random intercept were used for these analyses, multilevel linear regression models with continuous variables and multilevel multinomial regression models with categorical dependent variables [33]. These analyses were carried out on perennial organizations only. The two points in time were handled in the analysis by using, as covariables in the regression models, 2003 scores, the outcome variables being the difference between pre- and post-reform scores. Statistical significance is expressed in the tables by p-values.

## Results

### Organizational change

**Figure 2:**
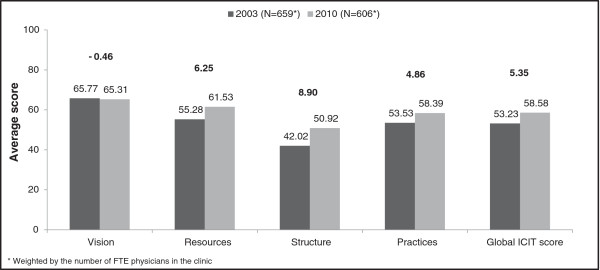
Average ICIT scores by domain, all clinics, 2003 and 2010*.

Organizational change was measured by the differences in ICIT scores between the 2005 and 2010 surveys. As explained earlier, the scores used for calculating the differences are adjusted for FMGs accredited prior to 2005. Hence, the post-reform period extends from 2003 to 2010. Figure 2 presents ICIT scores by domain and globally, as well as their differences between 2003 and 2010. The ICIT score rose by 5.35 on a 100-point scale with much of the increase due to the “structure” and “resources” domains.

**Figure 3:**
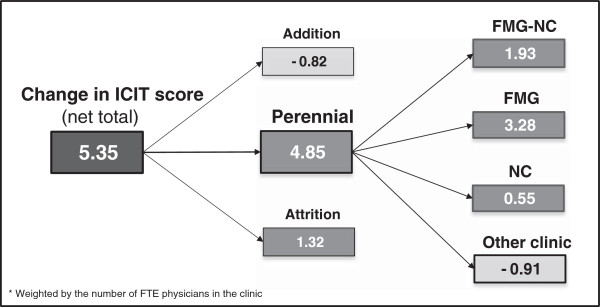
Three components of ICIT score change between 2003 to 2010*.

As mentioned earlier, the difference in ICIT scores is due to three effects related to perennial, added and closed organizations. Figure 3 details the contribution of the three ICIT score components to the 5.35 increase. More than 90% of this change is attributable to the effect of perennial organizations present both in the 2005 and 2010 surveys (4.85 points). The second most important contribution to the increase is due to the attrition effect of organizations that shut down between the 2005 and 2010 surveys or ceased to exist in their current form. Paradoxically, this attrition effect was positive (1.32), because most of these organizations were small and had low ICIT scores in 2005. Finally, organizations that opened between the 2005 and 2010 surveys had a negative effect of -0.82 (although expressed as an “addition” effect) on the ICIT score.

A more detailed examination of perennial organizations revealed a positive joint contribution of 5.76 points of FMGs and NCs, to the score of perennial organizations (Figure 3). “Other clinics” contributed negatively to overall change, reducing the ICIT score by 0.91 point.

Table 1 shows ICIT scores by domains and type of PHC organizations, at the onset of the reform and in 2010. The major increase in the global ICIT score noted was for PHC organizations having a double FMG-NC status. In second place, FMGs and NCs increased their score substantially. Finally, the score for other clinics slightly increased. Among other clinics, those having expressed the desire to become FMG or NC had a higher ICIT score in 2003 as well as in 2010, than those having not expressed this desire. Their ICIT score remained stable over the years.

**Table 1:** Average ICIT score by domain and by type of perennial organizations, 2003 to 2010

	**Vision**		**Resources**		**Structure**		**Practices**		**Global ICIT score**	
	**2003**	**2010**	**2003**	**2010**	**2003**	**2010**	**2003**	**2010**	**2003**	**2010**
FMG-NC (n=18*)	58,21	55,78	66,03	80,74	41,34	79,23	57,12	74,34	56,05	74,34
FMG (n=86*)	76,23	73,29	63,92	71,31	55,35	73,82	55,40	71,05	60,89	72,11
NC (n=17*)	53,29	57,94	67,47	75,65	53,51	71,25	62,57	64,67	60,37	68,11
Clinic without FMG or NC status that expressed the desire to become FMG or NC (n=72*)	69,34	70,28	53,26	57,89	40,04	40,34	57,23	53,59	54,06	54,26
Clinic without FMG or NC status that did not express the desire to become FMG or NC (n=348*)	64,47	62,76	48,87	50,17	34,62	29,75	50,72	46,94	48,62	46,28

*Weighted by the number of FTE physicians in the clinic.

### Explanatory factors of change

**Figure 4:**
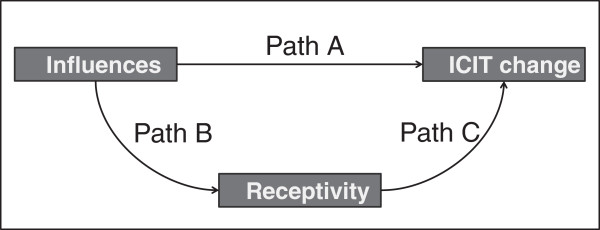
Analytical framework.

Figure 4 presents the analysis framework and shows the relationships between ICIT and “influences” whereas “receptivity” acts as a mediator variable [34]. Perceived influences of HSSC (coercive), professional associations (normative) and other clinics (mimetic), at both the organizational and local network levels, are posited to act directly on ICIT and indirectly through “receptivity”. Analyses in this section include perennial organizations only.

Table 2 presents results of the multilevel analysis for path A showing the influence on the ICIT change of HSSC (coercive), professional associations (normative), and other clinics (mimetic), as perceived by PHC organizations. Coercive and mimetic factors exert a positive influence on ICIT score change; normative factors do not influence ICIT score change. All corresponding local network variables are not significant.

**Table 2:** Factors associated with ICIT score change (100-point scale) excluding receptivity (Path A), 2003 to 2010 – Multilevel linear regression† (n = 541*)

**Level**	**Variables**	**Coeff.**	**p-values**
**Organizational**	**Coercive influence** (ref.: Negative or no action)		
	Positive	**3,590**	0,013
	**Normative influence** (ref.: Negative or no action)		
	Positive	**2,214**	0,179
	**Mimetic influence** (ref.: Negative or no action)		
	Positive	**8,371**	0,000
**Contextual**	**Coercive influence** (proportion of clinics judging positive HSSC actions)	**0,080**	0,137
	**Normative influence** (proportion of clinics judging positive the influence of professional associations)	**-0,036**	0,544
	**Mimetic influence** (proportion of clinics judging positive the influence of PHC organizations)	**0,033**	0,421

†Adjusted for ICIT score (2003), proportion of the population aged 65 and over, proportion of the population with low income, number of general practitioners per 100,000 inhabitants and avoidable mortality rates in the HSSC territory.

*Weighted by the number of FTE physicians in the clinic.

The direct effect of “influences” on ICIT change fades away when receptivity variables are entered in the model, except for coercive influence which still has a significant, though diminished effect (Table 3). This full model shows the effects of receptivity factors and influence variables (paths A + C) on ICIT change. When compared to the category of those that did not want to become FMG or NC, receptivity factors emerge as very significant, in the following order of importance: FMG-NC, FMG, NC and finally organizations that expressed their desire to become FMG or NC.

**Table 3:** Factors associated with ICIT score change (100-point scale) including receptivity (Paths A and C), 2003 to 2010 – Multilevel linear regression† (n = 541*)

**Level**		**Coeff.**	**p-values**
**Organizational**	**Coercive influence** (ref.: Negative or no action)		
	Positive	**2,192**	0,044
	**Normative influence** (ref.: Negative or no action)		
	Positive	**2,266**	0,096
	**Mimetic influence** (ref.: Negative or no action)		
	Positive	**1,605**	0,093
	**Receptivity** (Ref.: Clinic without FMG or NC status that did not express the desire to become FMG or NC)		
	Clinic without FMG or NC that expressed the desire to become FMG or NC	**4,027**	0,000
	NC	**10,056**	0,000
	FMG	**16,116**	0,000
	FMG-NC	**20,270**	0,000
**Contextual**	**Coercive influence** (proportion of clinics judging positive HSSC actions)	**0,081**	0,028
	**Normative influence** (proportion of clinics judging positive the influence of professional associations)	**-0,124**	0,012
	**Mimetic influence** (proportion of clinics judging positive the influence of PHC organizations)	**0,038**	0,244
	**Receptivity** (Proportion of receptive clinics)	**-0,005**	0,846

†Adjusted for ICIT score (2003), proportion of the population aged 65 and over, proportion of the population with low income, number of general practitioners per 100,000 inhabitants and avoidable mortality rates in the HSSC territory.

*Weighted by the number of FTE physicians in the clinic.

Path B was also explored to determine the effect of “influences” on receptivity, using a multinomial multilevel regression analysis (Table 4). Mimetic influences are significantly and strongly associated with higher degree of receptivity for FMGs, NCs, and to a lesser degree for other clinics having expressed the desire to become FMG or NC. Coercive factors are positively associated with receptivity for FMG-NC, FMG and NC.

**Table 4:** Factors associated with receptivity to become NC, FMG or FMG-NC (Path B), 2010 - Multilevel multinomial logistic regression† (n = 541*)

**Level**	**Variables**	**OR**	**p-values**
**Clinic without FMG or NC status that expressed the desire to become FMG or NC**			
**Organizational**	**Coercive influence** (ref.: Negative or no action)		
	Positive	**0,638**	0,363
	**Normative influence** (ref.: Negative or no action)		
	Positive	**1,495**	0,313
	**Mimetic influence** (ref.: Negative or no action)		
	Positive	**3364**	0,007
**NC, FMG or FMG-NC**			
**Organizational**	**Coercive influence** (ref.: Negative or no action)		
	Positive	**1,766**	0,030
	**Normative influence** (ref.: Negative or no action)		
	Positive	**1.366**	0,337
	**Mimetic influence** (ref.: Negative or no action)		
	Positive	**12,533**	0,000

†Adjusted for ICIT score (2003), proportion of the population aged 65 and over, proportion of the population with low income, number of general practitioners per 100,000 inhabitants and avoidable mortality rates in the HSSC territory.

*Weighted by the number of FTE physicians in the clinic.

**Reference:** Clinic without FMG or NC status that did not express the desire to become FMG or NC.

## Discussion

Our study results show that much of the change in the ICIT score from 2003 to 2010 was due to the “perennial” effect of PHC organizations that were present both in the 2005 and 2010 surveys. Given that the number of organizations in this category is substantial, it is not surprising that they contributed the most to overall organizational change. Paradoxically, the “addition” effect of organizations that opened after 2005 contributed negatively to the ICIT score change and those that closed during that period (attrition effect) contributed positively. In the former case, these organizations opened during a period of emergence of FMGs and NCs, when an important ICIT score increase was observed. It may have been difficult for an organization that opened during that period to rise to this level so soon after its inception. Therefore we can hypothesize that these organizations would have needed more time to fully develop and meet accepted standards. This could also suggest that the development of new clinics is not an area covered by the current PHC reform, which concentrates solely on established clinics accepting to conform to the accreditation process, to receive governmental support. In the second case, most organizations that closed were small or solo clinics and consequently had low ICIT scores at the time of the 2005 survey. This again is not surprising given their rudimentary form of organization and the fact that many of their owners might have been thinking about closing, therefore making organizational changes less relevant. Hence, aside from perennial organizations whose effect was more in line with emerging models of FMGs and NCs, the improvement in ICIT scores was in part due to a “natural selection” effect of clinics that closed, and this effect was mitigated by clinics that opened after the 2005 survey.

This result suggests that change in ICIT score could be associated to both the trend in evolution of organizations as well as to the reform policies designed to support the implementation of FMGs and NCs. Further analyses showed that this effect was not direct, but mainly mediated by receptivity of PHC organizations. Analysis of the relationship between “influences” and “receptivity” variables revealed that the most important factors explaining this relationship are “mimetic”, related to the influence of other exemplar PHC organizations. This mimetic influence played a major role mainly for FMG-NC, FMG, NC, and to a lesser, though significant degree, for PHC clinics wishing to become FMG or NC. Perceived influence of HSSCs did not play an important positive role.

Normative influence of professional association was not significantly associated with receptivity. This could mirror the reluctance and sometimes the opposition of professional associations to support the FMG movement, namely by promoting the alternative form of NC in the Montréal region [1].

To sum up, changes have been noted between 2003 and 2010, in relation to reform policies initiated at the provincial level in the early 2000s. The most important changes were observed in the “structure” and “resources” domains of the ICIT score and much less in the “practices” domain. This is easy to understand since the creation of FMG and NC has brought about resources and required modifications of structural arrangements. “Practices” always lag behind during reforms and are slower to develop. Vision remained stable.

Much of the change is due to receptivity factors which, in addition to their direct effect, mediate the slight influence of HSSCs on variations in ICIT score. This is not to say that the reform policies did not have an influence. Obviously, the establishment of FMGs was initiated by the Ministry of Health and social services and NCs were strongly supported by the Regional Montréal’s Regional Agency. However, at the local network level, the supportive role of HSSCs in the implementation of FMG and NC was not perceived as major. More importantly, the mimetic influence of other clinics provided an incentive and support for PHC clinics that became or expressed the desire to become FMG or NC. This result reflects a “bottom-up” strategy of change.

Viewed together, these results suggest that a decreed “top-down” reform was instrumental and an obvious prerequisite for initiating change in the healthcare system but it still needed to be implemented in a context of receptivity to change and the presence of “local champions”, advocating for the new models and demonstrating their feasibility and desirability.

### Limitations and strengths

Our study has some limitations. One is the way we measured receptivity. Except for the fourth and the fifth categories of clinics that expressed their desire or disinterest to become FMG or NC, the three first categories were only proxies for receptivity, since we did not have a direct measure of receptivity or readiness of these organizations before they became FMG or NC. It can be reasonably argued that clinics that became FMG or NC, were more receptive than those in the two last categories. Although there is an obvious link between receptivity as it is constructed and organizational change, there is a conceptual distinction between the two based on the notion of organizational change. As explained earlier, the process of organizational change initiated by the accreditation procedure did not stop there but improved over time, thus bringing about further changes, particularly in terms of structures and resources.

Secondly, although the design enables comparisons of the same organization at two points in time-corresponding approximately to a before-after scheme - it does not allow for comparisons with a control group where the reform has not been implemented. In this sense, it corresponds to a “natural experiment” rather than a true experimental design. However, in this case, variations in the influence exerted among HSSCs compensate to some extent for the lack of a comparison group.

A third limitation is the response rate and the probabilistic imputation method that we used for non-respondents. This method was applied to strata whose construction was based on region, size and type of organizations. It is a more rigorous method than simply randomly selecting subjects among respondents to replace non-respondents, particularly when response rates are acceptable, as they are in our study. As Haziza puts it, it is preferable to use imputation rather than do the analyses on incomplete data that may not be representative of the population [28]. In other words, the non-response bias is greater with non-response than with imputation and this is particularly important when inference must be made to the system’s effect of a reform. To test the extent of this possible bias, we performed a sensitivity analysis by introducing the variable “imputation” into the full multilevel analysis model presented in (Table 3). This variable failed to reach statistical significance. We also conducted a statistical analysis on respondent organizations only, and the results obtained were very similar to those presented, with a slight loss of statistical power.

A fourth limitation is that key informants who responded to the questionnaire may have been different in 2005 and 2010, thus introducing a possible respondent’s bias. In the end, the figure was relatively low (27% of responding organizations). In addition, we compared the 26 organizational variables among organizations that had two different respondents and among organizations with the same respondents in 2005 and 2010. We matched them using the same stratification variables as for the imputations, namely region size and type of organization. There were no significant differences between two different respondents and a single respondent on most of the variables. We should recall that the questions used to construct ICIT refer to factual information and, as such, are less likely to be affected by perception biases. As for imputation, we did a sensitivity analysis by introducing the variable “respondent” (same = 0, different = 1) into the full multilevel model presented in (Table 3). This variable did not come out as statistically significant.

Like many studies on organizational change, this article focuses on the content of change and on contextual factors explaining change, but not on its outcomes and consequences. In the larger study of which this article is a part, population surveys were conducted concomitantly with organizational surveys in the two regions in 2005 and 2010, with nominal linkage of the respondents to their regular source of care. This enabled us to link population and organizational data. Therefore, it is possible to assess the outcomes of change on the population in terms of services utilization, unmet needs and experience of care (accessibility, continuity, comprehensiveness and responsiveness). This important area of research will be examined in further publications.

Our study also has singular strengths. The main one is the use of a quantitative measure of ICIT change that enabled us to assess the likely effect of the reform both globally and by domain. Likewise, identification of perennial organizations as well as of those that emerged or disappeared between 2005 and 2010 made it possible to partition the global effect into perennial, addition and attrition effects. Finally, we analyzed organizational change longitudinally, by comparing two time points over a seven-year period (2003-2010).

## Conclusion

This article reports the results of a study aimed to assess the impact of two components of a healthcare policy put in place in the early 2000s. Specifically, the objectives of the study were to assess the evolution of PHC organizations between 2003 and 2010 and to assess the contribution of contextual and local factors in reform implementation. Our findings reveal only a modest organizational change at the population level with regard to the policy initiated by the Ministry. It must be kept in mind that the time horizon is relatively short to observe detectable changes. In addition, the secular downward trend observed for PHC organizations not directly touched by the reform was considerably offset by the changes recorded by new forms of organizations created by the same reform. We can easily conjecture that the overall situation would have been worse without these changes. What also emerges from the results reported in this article is that decreed policy change initiated by the Ministry was a necessary but insufficient condition for inducing change in the context of reform policy. The support given locally by HSSCs does not appear to have been very substantial, at least as perceived by PHC organizations. What seemed to have been much more important, at the local level, was the influence of other PHC organizations that acted as models of change to be imitated by other clinics (mimetic effect). This suggests that a “top-down” strategy initiated by the Ministry is more likely to yield success if coupled and reinforced with a local “bottom-up” strategy that rests on the influence PHC organizations can exert on each other rather than on the coercive influence of bureaucratic structures. These findings highlight the need for a participatory approach in implementing health care policies that concern the local level.

## Competing interests

The authors declare that they have no competing interests.

## Authors’ contributions

RP participated to all the stages of the study and drafted the manuscript. RB and AC coordinated the organizational part of the study and helped to draft the manuscript. JFL and SP participated in the design, the conduct of the study and drafting of the manuscript. MF advised on and supervised the statistical analysis performed by AP. All authors read and approved the final manuscript.

## Pre-publication history

The pre-publication history for this paper can be accessed here:

http://www.biomedcentral.com/1472-6963/14/229/prepub

## Supplementary Material

Additional file 1 **The organizational questionnaire contains all the questions completed by the respondent for every PHC organization.** It pertains to various aspects of this organization, such as vision, structure, resources and practices. The last section deals with the reorganization of PHC services. Click here for file

Additional file 2 List of variables* used to construct the index of conformity to an ideal type (ICIT). Click here for file

Additional file 3 List of explanatory variables. Click here for file

Additional file 4 List of control variables. Click here for file
