# Supplementary material for: Impact of Québec’s healthcare reforms on the organization of primary healthcare (PHC): a 2003-2010 follow-up
Source: BMC Health Serv Res. 2014 May 21;14:229. doi: 10.1186/1472-6963-14-229 (PMC4035759; doi:10.1186/1472-6963-14-229)
Supplement: Additional file 2 — List of variables* used to construct the index of conformity to an ideal type (ICIT). [file 1472-6963-14-229-S2.pdf]

Variables	Coding
**Domain: Vision**	
People targeted by the clinic	2) Population of the territory<br>1) Clients<br>0) Individuals who present
Organizational priority	2) Continuity of care<br>0) Accessibility
Importance of teamwork	2) + Important<br>1) +/- Important<br>0) - Important
Shared values regarding the clinic's objectives	2) High<br>1) Moderate<br>0) None
**Domain: Resources**	
Number of general practitioners working in the clinic	2) 6 and more<br>1) 2 to 5<br>0) 1
Roles and functions of the nurses in the clinic	2) Expanded role<br>1) Limited role<br>0) No nurse in the clinic
Presence of other professionals/specialists in the building in which the clinic is located	2) Specialists and other health professionals<br>1) Specialists or other health professionals<br>0) None
Number of information technologies in the clinic	2) 2 information technologies or more<br>1) 1 information technology<br>0) None
Proportion of general practitioners working 26 hours/week or more in the clinic	2) 100% (all)<br>1) 50% to 99%<br>0) 49% and less
Sharing of administrative resources among general practitioners in the clinic	2) High<br>1) Moderate<br>0) None
Availability of a technical platform in the building where the clinic is located	2) Radiology with or without blood samples taking<br>1) Blood samples taking only<br>0) None
**Domain: Structure**	
Sources of funding	2) Public or mixed<br>0) Private
Medico-administrative matters managed by	2) Administrative manager<br>1) Physicians<br>0) No management
Care coordination among clinic professionals	2) Pre-established care protocols<br>1) Informal or ad hoc exchanges only<br>0) Little or no coordination
Collaboration with other PHC clinics	2) High<br>1) Moderate<br>0) None
Collaboration with hospitals	2) High<br>1) Moderate<br>0) None
Participation of physicians to regional committees	2) High<br>1) Moderate<br>0) None
**Domain: Practices**	
Coverage extended to evenings and weekends	2) High<br>1) Moderate<br>0) None
Availability in emergency situations for regular patients	2) High<br>1) Moderate<br>0) None
Participation of the clinic to a healthcare access network	2) Yes<br>0) No
Consultation mode prevailing at the clinic	2) Mixed<br>1) More by appointment<br>0) Less by appointment
Scheduled length of time for consultations	2) Long<br>1) Moderate<br>0) Short
Scope of diagnostic, therapeutic and preventive services offered in the clinic	2) Broad<br>1) Moderate<br>0) Narrow/None
Systematic management of chronic diseases in the clinic	2) Yes<br>0) No
Sharing of clinical activities among general practitioners in the clinic	2) High<br>1) Moderate<br>0) None
Number of mechanisms to assess and maintain competence	2) High<br>1) Moderate<br>0) None
**Index of conformity to an ideal type** (score expressed on a 100-point scale)	

* Detailed construction of the variables is available from authors upon request.
