# Supplementary material for: Impact of Québec’s healthcare reforms on the organization of primary healthcare (PHC): a 2003-2010 follow-up
Source: BMC Health Serv Res. 2014 May 21;14:229. doi: 10.1186/1472-6963-14-229 (PMC4035759; doi:10.1186/1472-6963-14-229)
Supplement: Additional file 3 — List of explanatory variables. [file 1472-6963-14-229-S3.pdf]

| Indicators | Level 1: Organizational | Level 2: Contextual |
|---|---|---|
| **Receptivity** | **Label**: Receptivity to become Family Medicine Group (FMG) or Network clinic (NC)<br>0) Clinic without FMG or NC status that did not express the desire to become FMG or NC<br>1) Clinic without FMG or NC status that expressed the desire to become FMG or NC<br>2) NC, FMG or FMG-NC (double status) | **Label**: Proportion of clinics that are FMG-NC, FMG, NC or express the desire to become FMG or NC in each HSSC territory |
| **Coercive influence** | **Label**: Perceived effect on the clinic of the actions taken by the HSSC<br>0) Negative<br>1) No action<br>2) Positive | **Label**: Proportion of clinics judging positive HSSC's actions in each HSSC territory |
| **Normative influence** | **Label**: Perceived effect on the clinic of professional associations<br>0) Negative<br>1) No action<br>2) Positive | **Label**: Proportion of clinics judging positive the influence of professional associations in each HSSC territory |
| **Mimetic influence** | **Label**: Perceived effect on the clinic of other PHC organizations (introduction of FMG or NC in the region and exemplary practices)<br>0) Negative<br>1) No action<br>2) Positive | **Label**: Proportion of clinics judging positive the influence of other PHC organizations in each HSSC territory |
