# Supplementary material for: Impact of Québec’s healthcare reforms on the organization of primary healthcare (PHC): a 2003-2010 follow-up
Source: BMC Health Serv Res. 2014 May 21;14:229. doi: 10.1186/1472-6963-14-229 (PMC4035759; doi:10.1186/1472-6963-14-229)
Supplement: Additional file 4 — List of control variables. [file 1472-6963-14-229-S4.pdf]

Indicators	Level 1: Organizational
**Index of conformity to an ideal type**	**Label:** Index of conformity to an ideal type adjusted for FMGs accredited prior to 2005<br>*Score on a 100-point scale*
**Indicators**	**Level 2: Contextual**
**Age of the population**	**Label:** Proportion of the population aged 65 and over in each territory<br>*Expressed as a percentage*<br>**Definition:** Demographic projections of population by age (ISQ, 2010)
**Income of the population**	**Label:** Proportion of the population with low income in each territory<br>*Expressed as a percentage*<br>**Definition:** The level of low income is defined as "income levels under which it is estimated that families or unattached individuals spend 20% more than the average of their income on food, housing and clothing" (Statistics Canada, 2007)
**Health of the population**	**Label:** Annual avoidable mortality rate per 100,000 inhabitants in each territory<br>*Rate per 100,000 inhabitants*<br>**Definition:** All causes of death considered avoidable. Standardized rates for the age structure (INSPQ, 2005 to 2007)
**Resources of the HSSC territory**	**Label:** Number of general practitioners per 100,000 inhabitants in each territory<br>*Rate per 100,000 inhabitants*<br>**Definition:** Number of general practitioners per 100,000 inhabitants (MSSS, 2009)
